# Potential decline in the distribution and food provisioning services of the mopane worm *(Gonimbrasia belina)* in southern Africa

**DOI:** 10.21425/F5FBG59408

**Published:** 2023-06

**Authors:** David Y. Shen, Henry Ferguson-Gow, Vivienne Groner, Thinandavha C. Munyai, Rob Slotow, Richard G. Pearson

**Affiliations:** 1 Centre for Biodiversity and Environment Research, Department of Genetics, Evolution and Environment, University College London, London, WC1E 6BT, United Kingdom; 2 School of Life Sciences, University of KwaZulu-Natal, Private Bag X01, Scottsville, 3209, South Africa

**Keywords:** biotic interactions, ecosystem services, entomophagy, species distribution models

## Abstract

The mopane worm *(Gonimbrasia belina)* is an edible insect distributed across southern Africa. As a culturally important source of food, the mopane worm provides nutrition, livelihoods and improves wellbeing for rural communities across its range. However, this is strong evidence that insect populations are declining worldwide, and climate change is likely to cause many insect species to shift in their distributions. For these reasons, we aimed to model how the ecosystem service benefits of the mopane worm are likely to change in the coming decades. We modelled the distribution of the mopane worm under two contrasting climate change scenarios (RCPs 4.5 and 8.5). Moreover, given that the mopane worm shows strong interactions with other species, particularly trees, we incorporated biotic interactions in our models using a Bayesian network. Our models project significant contraction across the species’ range, with up to 70% decline in habitat by the 2080s. Botswana and Zimbabwe are predicted to be the most severely impacted countries, with almost all habitat in Botswana and Zimbabwe modelled to be lost by the 2080s. Decline of mopane worm habitat would likely have negative implications for the health of people in rural communities due to loss of an important source of protein as well as household income provided by their harvest. Biogeographic shifts therefore have potential to exacerbate food insecurity, socio-economic inequalities, and gender imbalance (women are the main harvesters), with cascading effects that most negatively impact poor rural communities dependent on natural resources.

## Introduction

Biodiversity and associated ecosystem services play an important role in influencing and improving human wellbeing (Corvalán et al. 2005, Haines-Young et al. 2010, Brondizio et al. 2019). The value of provisioning services such as food and fuels (Corvalán et al. 2005) can be especially high for poorer households, (Kenter et al. 2011, Shackleton 2021). Food provisioning services, in particular, can have an important role in improving food security year round in areas strongly impacted by seasonal variation in food availability (Bharucha and Pretty 2010, Arnold et al. 2011). During the “lean season” (the time between the planting and harvesting of crops) wild harvested food sources can vastly improve the quality of life for rural communities (Arnold et al. 2011, Cruz-Garcia and Price 2014, Dragicevic 2017).

One important non-agricultural food are edible insects, which have been identified by the Food and Agriculture Organisation of the United Nations as a solution to reduce food insecurity (van Huis 2015). Insect consumption by humans (entomophagy) can have significant benefits for human health because insects contain high amounts of protein and minerals to help reduce undernutrition (Christensen et al. 2006, van Huis 2015, Nadeau et al. 2015, Hlongwane et al. 2020). The mopane worm is an example of an important wild food insect in southern Africa (van Huis et al. 2013, Hlongwane et al. 2021).

The mopane worm is the caterpillar form of the emperor moth *Gonimbrasia belina,* a widely distributed Saturniid found across and native to southern Africa. *G. belina* has historically provided an important source of nutrition and protein for local communities, who harvest the larvae in large numbers (Stack et al. 2003). These caterpillars are widely eaten across southern Africa, with over 80% of interviewed households in the Limpopo province of South Africa consuming *G. belina* at least 3-5 times a week (Kozanayi and Frost 2002, Stack et al. 2003, Obopile and Seeletso 2013, Makhado et al. 2014, Baiyegunhi and Oppong 2016, Hlongwane et al. 2021). For example, in Botswana *G. belina* remains a culturally important food despite the overall decline in entomophagy (Obopile and Seeletso 2013). The significant protein content of *G. belina* is particularly important given that Africa has the lowest protein intake per capita in the world (Illgner and Nel 2000, Schönfeldt and Hall 2012). One hundred grams of dried *G. belina* can provide 76% of a human’s daily protein requirement, as well as many necessary vitamins and minerals (Christensen et al. 2006, Potgieter et al. 2012, Makhado et al. 2014). This is particularly important in southern Africa, where many households commonly suffer from some form of food insecurity (De Cock et al. 2013, Baiyegunhi and Oppong 2016).

*G. belina* caterpillars feed primarily, though not exclusively, on the mopane tree *(Colophospermum mopane),* from which it derives its name (Ditlhogo 1996). Adult moths emerge from pupae and deposit eggs on leaves of *C. mopane* and other host trees during the first few weeks of the rainy season, around October. Occasionally, if there is enough rainfall during February, the caterpillars will show a second, smaller outbreak in April (Taylor and Moss 1982, Ditlhogo 1996). Once hatched, the caterpillars undergo a 4,000 fold increase in body mass over the course of 6 weeks (Gaston et al. 1997). The caterpillars are usually harvested around December to January, during the lean season, by which time the caterpillars have reached their largest and final developmental stage before they pupate (Ditlhogo 1996). Those that are not harvested at this stage bury themselves underground to pupate.

Harvesting caterpillars provides nutrition and generates a valuable source of income for many rural communities (Illgner and Nel 2000, Kozanayi and Frost 2002, Stack et al. 2003, Hlongwane et al. 2021). Income from selling *G. belina* can substantially improve the wellbeing of a rural household, as income is a major determinant of food security (Ghazoul et al. 2006, De Cock et al. 2013). Baiyegunhi and Oppong (2016) reported that 63% of harvested *G. belina* in Limpopo, South Africa, are sold in local markets. It is therefore apparent that *G. belina* provides an important safety-net to the wellbeing of poor rural communities in southern Africa. Understanding how the distribution of *G. belina* could be influenced by climate change is therefore of importance in understanding how food security and community health is likely to be impacted.

There have been significant observed insect declines around the world, and several studies predict further declines under climate change (Maes et al. 2010, Hallmann et al. 2017, Sánchez-Bayo and Wyckhuys 2019). Given that *G. belina* consumes the leaves of only a limited number of tree species, particularly *C. mopane,* the species may be especially vulnerable to decline. Many plant species are expected to be unable to shift their distributions quickly enough to keep pace with climate change (Corlett and Westcott 2013). The longer lifecycle of trees may make them slower to shift their distribution with climate change compared to more mobile species or those with shorter lifecycles, leading to range contraction and fragmentation. Differences in the biogeographic responses of *G. belina* and its food tree species may therefore lead to range decline of *G. belina* and localised extinctions.

Insects that are closely co-adapted to their host plants may also have difficulty shifting their distribution due to an inability to switch to alternative food plants, whereas generalist species are more likely to switch hosts and thus be less negatively impacted by climate change (McKinney 1997, Warren et al. 2001, Colles et al. 2009, Pelini et al. 2010, Davey et al. 2012). *G. belina* is not an exclusive herbivore of C. mopane, so it may have some ability to switch to other species. Mughogho and Munthali (1995) found that while *G. belina* consumed several other tree species, there were apparent differences in preference. This preference may mean that *G. belina* has the potential to switch to other, less preferable food trees. However, if the less preferable species represent poorer quality food sources, this may negatively impact on how many *G. belina* individuals could be supported.

Climate change is predicted to negatively alter ecosystem services (Mooney et al. 2009, Groner et al. 2022). These impacts are likely to be severe for southern Africa; for instance a review by Serdeczny et al., (2017) predicted a significant increase in undernutrition in southern Africa under climate change, leading to greater risk of negative secondary health consequences. Negative impacts of climate change on crop yield in southern Africa may lead to increased reliance on wild species as food resources (Zinyengere et al. 2013). However, a study by Ndlovu et al.(2019) found that harvests of *G. belina,* as a proxy for population, had collapsed by 98% from 2007 to 2016 in the Mangwe district of Zimbabwe. Under further climate change, collapses in harvest and population may be more likely. Therefore, understanding the impact of climate change through biogeographic modelling can help us anticipate further declines.

Species distribution models (SDMs) can be used to predict future distribution trends under scenarios of environmental change (Elith and Leathwick 2009, Peterson et al. 2011). SDMs combine known occurrence records with abiotic variables to build statistical models of the abiotic niche of a species. This is then used to identify areas in a landscape that have similar environmental conditions to where the species is known to occur (Guisan and Zimmermann 2000, Elith and Leathwick 2009). A well-known limitation of this method is that biotic interactions are typically ignored, yet such interactions influence species’ distributions and are an important component of a species’ ecological niche (Pearson and Dawson 2003, Peterson et al. 2011, Wisz et al. 2013). For example, the presence of *G. belina* host trees is likely to positively impact the occurrence likelihood of *G. belina,* and predator pressure from birds and parasitoid wasps are likely to negatively impact occurrence likelihood (Price 1992, Styles 1995, Ditlhogo 1996, Gaston et al. 1997). Excluding biotic interactions from SDMs is thus expected to lead to less accurate predictions (Araujo and Luoto 2007, Wisz et al. 2013) Previous studies have used several methods to incorporate biotic interactions in SDMs and have demonstrated improved model performance (e.g., Fordham et al., 2013; Heikkinen et al., 2007; Palacio and Girini, 2018; Pellissier et al., 2013. Here we apply the method developed by Staniczenko et al.(2017) that uses Bayesian networks to enable the ‘knock-on’ impacts of biotic interactions to be captured Our study represents an early adoption of these promising, next-generation models. Predictions are then used to understand the potential changes in *G. belina* food provisioning services and how these may impact food insecurity, livelihoods and wellbeing.

## Materials & Methods

All analyses were conducted using R 4.0.3 in RStudio 1.4 (R Core Team 2020, RStudio Team 2021).

### Study site

**Figure 1:**
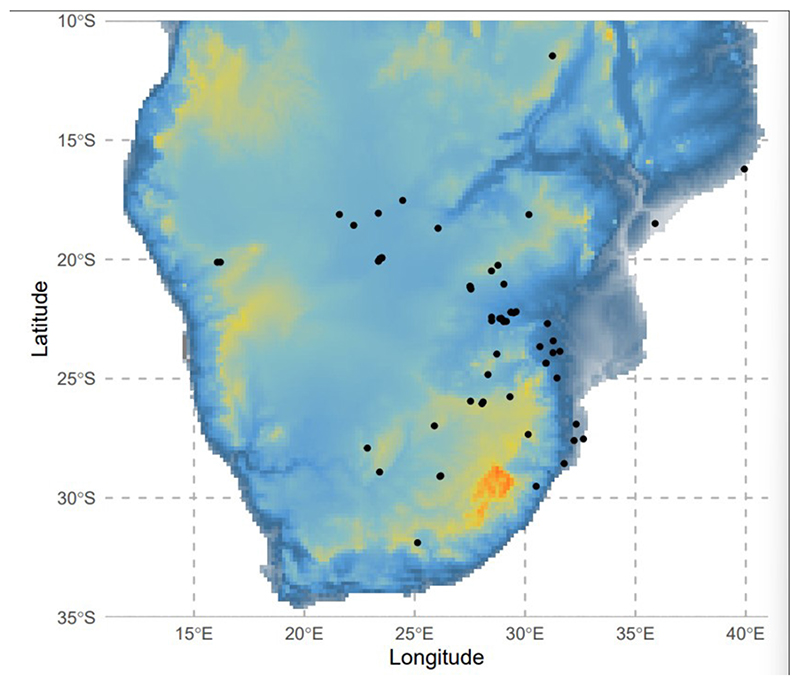
Study region in southern Africa used to construct SDMs. Black dots represent occurrence records of *Gonimbrasia belina,* overlaid on a topographical map of southern Africa.

Analysis was conducted in the native range of *G. belina* in southern Africa (Fig. 1). The bounding box for modelling encompassed the vast majority of *G. belina* occurrence records. This ensured that absences in distribution are largely caused by ecological filtering, rather than dispersal limitation, allowing better model prediction (Anderson and Raza 2010).

### Constructing the Bayesian network of interactions

A literature review was carried out to identify species that interacted with *G. belina.* These included several predatory bird species, parasitoid wasps, competitive herbivores, and 13 food tree species (Mughogho and Munthali 1995, Styles 1995, Ditlhogo 1996, Gaston et al. 1997, De Nagy Koves Hrabar 2007, Akinnifesi et al. 2008, Potgieter et al. 2012). In total, 51 interacting species were identified (Supplementary Table SI).

The direction of interaction (positive or negative) was determined from the ecological relationship between the interacting species and *G. belina.* Species that potentially increased *G. belina* mortality, such as predation, parasitoids and resource competition were classed as negative interactors. This placed all the predatory bird species and parasitoid wasp species as negative interactors. Species that improved *G. belina* survival were classed as positive interactors, such as food sources or mutualistic relationships. All the known food tree species were thus classed as positive interactors.

**Figure 2:**
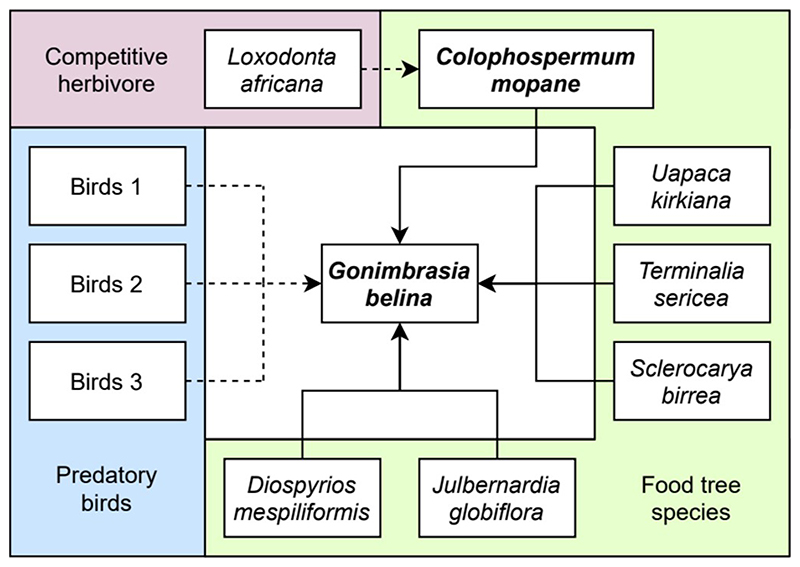
Bayesian network used to model the distribution of *G. belina.* Arrows drawn in dashed lines represent negative interactions, solid lines represent positive interactions. Species in bold represent focal species. Birds 1-3 represent the predatory bird functional groups.

Studies quantifying the strength of biotic interactions are limited, and we were only able to find one study regarding the food tree preferences of *G. belina* (Mughogho and Munthali 1995). However, this study lacks *C. mopane* as a food source, so is limited in its usefulness for calculating interaction strength. Therefore, we could only use binary positive or negative interactions in our models. A Bayesian network was constructed accordingly, centralising on *G. belina* (Fig. 2). To resolve this network, we compared the AND and OR resolution methods described by (Staniczenko et al. 2017). The AND method requires all dependencies of an interaction direction to be present for an effect, while the OR method only requires at least one dependency to be present for an effect. While the AND method performed marginally better with respect to its AUC score, it had minimal impact on the distribution. Therefore, we selected the OR method in our final model as we believed it better represented the biological interaction information, at the risk of potentially overestimating the strengths of biotic interactions.

### Data collection and preparation

In order to generate SDMs, species occurrence records were obtained from the Global Biodiversity Information Facility (GBIF) (GBIF.org 2022). Records reported as human observation, living specimen, or machine observation were used. Species were further filtered to those that had a minimum of 20 occurrence records. Less than 20 occurrence records would have increased the error of distribution models, and limited the number of k-fold cross validations that could be run (Pearson et al. 2007, Proosdij et al. 2016). In the end, 68 records were obtained for *G. belina.* The full table of all the identified interacting species and number of occurrence records obtained can be found in Supplementary Table SI. This criteria for records reduced the number of biotically interacting species included in the Bayesian network from 51 species to 31, including 24 bird species, 6 food tree species, and 1 herbivore. These occurrence records were cleaned using the CoordinateCleaner (version 2.0-18) package to remove erroneous or mislabelled data (Zizka et al. 2019). Occurrence records were tested for being exactly on country capital and centroid coordinates (indicating automatically generated coordinates), equal longitude and latitude, records one degree around the GBIF headquarters and other biodiversity institutions, records in the sea, and records with plain zeros. A total of 4,757 records failed these tests and were subsequently removed. Cleaned records were then rarefied to the climate data so there was only one occurrence record per grid cell.

Climate data were obtained from CHELSA for the 1979-2013 (here on referred to as ‘present’) period at a resolution of 30 arcseconds, which were aggregated to 2 arcminutes (Karger et al. 2017). Six bioclimatic variables were used: mean annual temperature, max temperature of the warmest month, minimum temperature of the coldest month, annual precipitation, precipitation of the driest month, and precipitation of the wettest month. These variables were chosen to incorporate the annual ranges of precipitation and temperature, and to account for the increased frequency of extreme events expected under climate change (Kusangaya et al. 2014, Dunning et al. 2018). Additionally, rainfall plays an important role in triggering the emergence of adult moths from pupae, necessitating the inclusion of rainfall and rainfall variation (Taylor and Moss 1982, Ditlhogo 1996).

For future predictions, we selected two contrasting representative concentration pathways (RCPs): 4.5 and 8.5. RCP 4.5 represents an intermediate climate scenario with atmospheric CO_2_ stabilising around 650 parts per million by the year 2100.

RCP 8.5 represents the worst case “no climate policy” scenario, with CO_2_ concentrations projected to exceed 1,300 parts per million by 2100. Bioclimatic data for these projections were obtained from the Coupled Model Intercomparison Project Phase 5 (CMIP5), modelled using HadGEM2-CC (Martin et al. 2011, Taylor et al. 2012). The original HadGEM2 datawere downscaled to 30 arcseconds using interpolation based on the reference CHELSA climatologies (Karger et al. 2017). This enabled the future species distribution predictions to maintain the same resolution as the present, to better show the predicted trends in change.

### Species distribution models

SDMs were implemented using three different algorithms: Bioclimate Envelopes (BIOCLIM), Generalised Linear Models (GLM), and Random Forests (RF). BIOCLIM and GLM SDMs were generated using the dismo (version 1.3-3) package, while RF SDMs were generated using the randomForest (version 4.614) package (Liaw and Wiener 2002, Hijmans et al. 2020). Three SDM algorithms were used to account for algorithm-based uncertainty (Pearson et al. 2006). BIOCLIM, GLM and RF models were used in particular as three contrasting algorithms: a simple climate envelope, a statistical regression model and a machine learning model respectively (Tsoar et al. 2007, Li and Wang 2013). We then combined the three models to create an ensemble (Araujo and New 2007) by combining the output rasters from each of the three algorithms. The habitat suitability value in each cell across all three algorithms was averaged and weighted by the algorithm’s calculated area under the received operator curve statistic (AUC) obtained from 4-fold cross validation. The AUC represents how well the output of each algorithm matches the original occurrence data, with scores ranging from 0.5 (random model) to 1 (representing perfect model performance). This process was repeated for the future climate data produce ensemble SDMs under climate change. The AUC of the ensemble SDM for *G. belina* was 0.812 with standard deviation of 0.051.

A threshold was applied to the ensemble model to convert the continuous probability of habitat suitability into binary ‘presence’ or ‘absence’ using the True Skill Statistic (Liu et al., 2013). We chose the TSS because of it is insensitivity to prevalence, decreasing the effect of spatial bias in our predictions (Allouche et al. 2006). While our analysis uses the TSS, we also plotted the results using a less restrictive Prevalence threshold to show the uncertainty caused by threshold selection.

### Resolving the Bayesian network

Preliminary tests identified that the time taken to resolve the Bayesian network increased rapidly with the number of links. The network quickly became incomputable beyond around 12 dependencies, despite highly parallelised processing. We used two methods to circumvent this practical limitation: (i) we grouped species into functional groups and then included the group as a node in the network; and (ii) we used a sub-network to model an important indirect interaction and then included the sub-network as a node in the main network.

We grouped species by function on the assumption that the type and direction of interaction within members of a functional group would be the same. For example, all predatory birds lethally consume *G. belina,* such that the outcome is the same regardless of the species attacking. We could have also condensed the tree species into functional groups, but we aimed to retain as much detail as possible in interactions between *G. belina* and its food trees because food trees have such a fundamental role in determining the distribution of herbivorous insects (Price 1992). We therefore condensed the 22 distribution model rasters for predatory birds into three by selecting the three species that had the highest habitat suitability in each cell, which was used as a proxy for the probability of occurrence (Birds 1-3 in Fig. 2). This was done based on the assumption that the species most likely to occur in an area would have the strongest biotic interaction with the focal species *G. belina.* By contrast, a species with very low occurrence probability in a cell was assumed to have minimal biotic interaction and was therefore excluded. This method meant that the species identity could change from cell to cell. Under the OR model, we assumed that the identity of an interacting species does not matter, but only whether the interaction direction is positive or negative (Staniczenko et al. 2017). Given this, we determined this method was an appropriate way to reduce the number of dependent species and allowed us to resolve the Bayesian network.

Our use of a sub-network was also to condense the impact of several species with indirect biotic interactions into a single raster. In our case, elephants are known to have indirect interaction with *G. belina* through resource competition, consuming the leaves of *C. mopane* (De Nagy Koves Hrabar 2007). This was modelled through a sub-network focusing on *C. mopane,* generating SDMs with each algorithm, then applying the Bayesian network to modify the distribution of *C. mopane* (Fig. 2). The modified distribution of *C. mopane* was then used as a prior for the Bayesian network focusing on *G. belina,* thereby incorporating the indirect interaction of the elephants, without increasing the number of dependencies.

The *C. mopane* SDM, SDMs of the other food tree species, and the three condensed predatory bird SDMs were then used as priors to resolve the Bayesian network focusing on *G. belina* (Fig. 2). This process was conducted to produce posterior distributions for each of BIOCLIM, GLM and RF SDMs. Four-fold cross validation was performed again to calculate the average AUC of each algorithm with the Bayesian network applied. This was used to combine the Bayesian SDMs from each algorithm into an average ensemble model weighted by AUC. After obtaining the ensemble model constructed using SDMs modified by the Bayesian network modified, a threshold was again applied detailed in section 2.4. AUC for the ensemble Bayesian network SDM for *G. belina* was 0.740 with standard deviation of 0.013.

## Results

**Figure 3:**
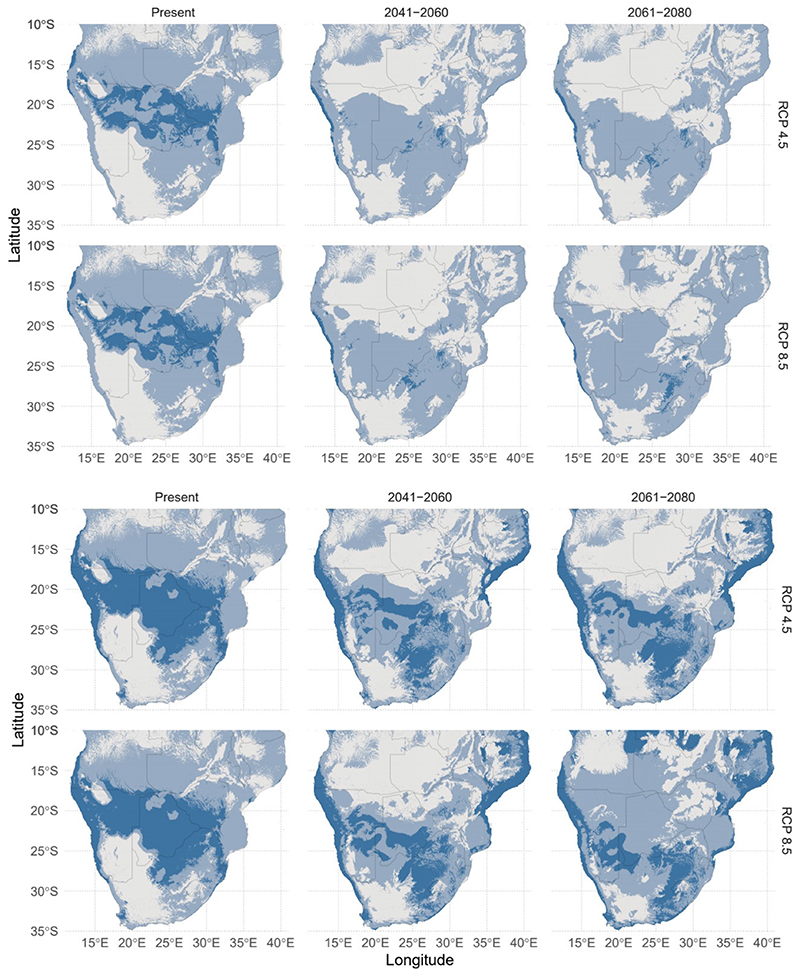
Top Bayesian network species distribution models of G. belina with threshold applied under RCP 4.5 and 8.5. Bottom Map of species distribution without Bayesian network, threshold applied, with future distribution predictions under RCP 4.5 and 8.5. Light blue represents a less stringent Prevalence threshold, dark blue represents a more stringent TSS threshold.

Comparing the difference between our Bayesian network and non-Bayesian network models, we find substantial differences in the number of cells predicted (Fig. 3). Overall, our Bayesian network model predicts around two times less suitable habitat distribution compared to the non-Bayesian network model under present climate, and more than ten times less suitable habitat under the different future climate scenarios. Predicted area differences under the Bayesian network and non-network models are summarised in Table 1.

Our results also show differences in habitat fragmentation between the network and no network models, as well as between different climate scenarios. Habitat can be seen to be much more disjointed under predicted climate scenarios with the network model (Fig. 3). Patch density, a measure of habitat fragmentation, was correspondingly found to be lower (Table 2). In the models with no Bayesian network applied, habitat was not observed to be as fragmented, and patch density was found to increase substantially (Table 2).

Using the Bayesian network models, we predict substantial reductions in the distribution of *G. belina* across southern Africa. Across the entire modelled region, 56% of *G. belina* habitat is predicted to be lost under the more moderate RCP 4.5 scenario by the 2080s (Fig. 3). This represents a decline in habitat area from around 649,000 km^2^, to around 285,000km^2^. While total habitat decline is greatest under RCP 8.5, with 65% of habitat area lost by 2080, the rate of habitat loss under RCP 4.5 is greater from the present period to 2040-2060, with 63% decline under RCP 4.5 compared to 52% under RCP 8.5. Full changes are summarised in Table 3. Climate change impacts on the distribution of *G. belina* also shows increasing fragmentation of the *G. belina* distribution.

In Botswana, Zimbabwe and South Africa, three countries that have widespread consumption of *G. belina,* substantial decline in *G. belina* distribution is predicted. However, each country shows differences in distribution change under climate change. Habitat loss is most pronounced in Botswana, given that present predictions suggest that 40% of the country is suitable habitat for *G. belina* (Fig. 3). Under RCP 4.5, 99% of the habitat is predicted to be lost by 2080, with only 16,700km^2^ remaining. Under RCP 8.5, 100% of modelled habitat is predicted to be lost (Table 3).

Similarly, major declines are predicted in southern Zimbabwe. Under RCP 4.5, *G. belina* habitat declines from covering the majority of southern Zimbabwe to just a few isolated pockets, disconnected from the main distribution. Under RCP 8.5, close to the entire *G. belina* habitat is projected to be lost, placing *G. belina* at risk of local extinction (Fig. 3). With this complete loss in suitable habitat, the corresponding food provisioning services would likely also disappear.

## Discussion

### Impact of climate change on G. belina distribution

Our models predict that the distribution of *G. belina* is likely to contract under future climate change, even under the more moderate scenario of RCP 4.5. These results support our initial hypothesis that climate change would lead to species distribution loss. While our models predicted significant distributional decline, by 2023 (10 years after the present climate data range) only around 10% of suitable habitat is predicted to be lost. Ndlovu et al. (2019) found that harvests of *G. belina,* as a proxy for population, had collapsed by 98% from 2007 to 2016 in the Mangwe district of Zimbabwe, so our modelled changes are in line with empirical observations.

The most significant loss of habitat can be seen at the tripoint of Botswana, Zimbabwe and South Africa (Fig. 3). Our modelling suggests that this area contains the most optimal habitat for *G. belina.* This loss may significantly and negatively alter the population of *G. belina* in the wider region through changes to the spatial dynamics. By acting as a source habitat, individuals may then migrate away from the source to sink habitats with less optimal conditions, sustaining populations which might otherwise be inviable (Dias 1996). This may enable the persistence of *G. belina* in the areas modelled to have lower habitat suitability. The loss of the most optimal habitat could have a disproportionately large impact on the population of *G. belina* in the wider area, and lead to more decline than modelled.

Some habitat gain in South Africa is predicted under both RCP scenarios modelled. This falls in line with previous studies that have identified poleward distribution shifts under climate change (Vanhanen et al. 2007). Emergence of new habitat would prevent the entire population from going extinct, as predicted in the northern areas. However, this presents the question of whether *G. belina* and its food trees can adequately track the moving suitable climate. Insects tend to have short lifespans and high mobility, enabling them to quickly respond to changing climates (Stange and Ayres 2010). By comparison, the food tree species have longer and sessile lifecycles, potentially making responding to changing climates more difficult. While many studies have predicted tree distributions to shift under climate change, the rate at which trees are able to shift is less well known (Hamann and Wang 2006, Monleon and Lintz 2015, Mathys et al. 2018). For example, Foden et al., (2007) found that the trailing edge of the *Aloe* tree distribution in Namibia was declining much more than the leading edge was expanding, leading to a net distribution loss. If the *G. belina* food trees show similar lag between the trailing and leading edges of their distribution, then an overall distribution decline would be expected. The distribution shift of *G. belina* would only be as fast as the slowest functional group shift, i.e. food trees. This may have the greatest impact under RCP 8.5, when the rate of climate change is higher. The distribution predictions for 2061-2080 under RCP 4.5 show striking similarity to that for 2041-2060 under RCP 8.5 (Fig. 3). If tree species are unable to shift their distribution south by 2080 under RCP 4.5, then it is even less likely that the tree species will be able to shift distribution with faster climate change under RCP 8.5. This would mean the 2041-2060 distribution under RCP 8.5 may be an overestimation of the future distribution. Unfortunately, these models were produced using only 6 of the 14 known food tree species for *G. belina,* due to the lack of occurrence records (supplementary Table SI). It may be possible that the tree species not included in our models remain largely constant in their future distribution, ensuring a constant food source for *G. belina.* With more occurrence records for these trees, it would then be possible to model their future distributions and evaluate the impact of climate change on *G. belina.* Further investigation into other food sources and food preferences for *G. belina* would be necessary to better model the relationships between food trees and *G. belina.*

The increased habitat fragmentation is also likely to lead to negative impacts on *G. belina.* Previous studies have shown that habitat fragmentation can lead to reduced insect herbivory (Valladares et al. 2006, Ruiz-Guerra et al. 2010, Rossetti et al. 2014). Reduced herbivory may mean that the much more fragmented distribution predicted by our models would likely be an overestimate of the future distribution. Conversely, Peter et al., (2015) found that forest fragmentation would lead to increased insect populations through the loss of insectivorous birds. Given that our Bayesian network incorporated insectivorous birds as predators, linking the distribution of birds back to the presence of trees may be an important additional interaction. Habitat fragmentation evidently has complex interactions with insect herbivory and insect predators. Field studies at the edges of present suitable *G. belina* habitat, especially in Zimbabwe, may provide a suitable approximation for the predicted fragmented habitat. This would aid in understanding how fragmentation would impact *G. belina* presence.

Fragmentation may also negatively impact *G. belina* through a decline in genetic diversity (Dixo et al. 2009, Vranckx et al. 2012). At present, *G. belina* shows a large amount of genetic diversity, despite its poor dispersal ability (Styles 1994, Greyling et al. 2001). Flighted adults only live for around 5 days, and are generally incapable of long distance flight (Styles 1994). Greyling et al. (2001) suggest that the high genetic diversity may be due to the stepping-stone model of gene flow (Peterson 1996). Habitat fragmentation may disrupt this process, and given the poor dispersal ability of *G. belina,* lead to even greater losses in genetic diversity than that which would otherwise be expected.

### Impact of *G. belina* decline on food security

The predicted loss of *G. belina* distribution would likely have a negative impact on food provisioning. At present, *G. belina* provides an important source of nutrition and income across southern Africa (Makhado et al. 2014, Kwiri et al. 2020). The losses predicted by our models would mean this important source of food and nutrition is lost for many people. As *G. belina* is highly seasonal, it is unclear what role it plays in the year-round health of rural communities; therefore, it is important that future studies evaluate how the loss of *G. belina* as a food and nutrient source may impact health.

The potential loss of *G. belina* may mean that other edible insects are harvested and consumed instead, but this is unlikely to be the case. While entomophagy was previously widespread across southern Africa, it is currently in decline (Illgner and Nel 2000, Obopile and Seeletso 2013). *G. belina* is particularly important as it remains the only insect still widely consumed, especially among young people (Obopile and Seeletso 2013). The decline in *G. belina* as an alternative source of protein is unlikely to be replaced by a similar edible insect, and may lead to increased consumption of bushmeat (Loibooki et al. 2002). This would have negative knock-on impacts on the populations and conservation status of many other wild species (Nasi et al. 2008). Ensuring that insects remain a sustainable, alternative source of protein not only has benefits for human health, but also alleviates consumption pressure on other wild species.

Another way *G. belina* contributes to food security is through trade, generating income and allowing households to buy food. Household income is known to be a major determinant of food security in this region (De Cock et al. 2013). Total *G. belina* trade has been valued at up to US$59 million per year, and can provide as many as 10,000 seasonal jobs (Makhado et al. 2014). For example, in the Limpopo region of South Africa, Baiyegunhi and Oppong (2016) found that households could double their monthly income through *G. belina* trade. As this region has particularly high levels of food insecurity, income provided by *G. belina* trade is especially important (De Cock et al. 2013). In Botswana, a similar trend would be expected, but on a far wider scale considering the wide *G. belina* distribution (Fig. 3). A survey in north-eastern Botswana found that selling *G. belina* allowed many to meet their basic needs, and the disappearances of *G. belina* have seriously challenged their finances (Selaledi 2012). *G. belina* harvesting in Botswana has largely shifted from a subsistence activity for household consumption to a commercial activity (Mogomotsi et al. 2018), so a significant loss in *G. belina* distribution could devastate the Botswanan *G. belina* trade. Given that much of the Botswanan *G. belina* harvest is exported to neighbouring countries such as Zimbabwe and South Africa, the decline of *G. belina* may lead to greater food insecurity both inside and outside of Botswana (Mogomotsi et al. 2018). Declines and even local extinctions of *G. belina* due to overexploitation has already been reported by harvesters, and this may be further compounded by climate change (Ghaly 2009). The economic impact of *G. belina* decline may therefore have more far-reaching impacts on food security and social wellbeing than its loss as a food and nutrient source.

### Evaluation of Bayesian network species distribution models

The results obtained using Bayesian networks to incorporate biotic interactions show substantial differences from the non-Bayesian network distribution models (Fig. 3), as expected (see review by Van der Putten et al. 2010). Our results incorporating biotic interactions show smaller distribution sizes overall, and much greater decline under climate change scenarios. Previous studies including biotic interactions in SDMs show that biotic interactions lead to improved model performance and fit (Heikkinen et al. 2007, Fordham et al. 2013, Pellissier et al. 2013, Staniczenko et al. 2017, Palacio and Girini 2018). Surprisingly, our results do not show improved fit in k-fold partitioning with the observed occurrence records; in fact, AUC reduced slightly (by 0.072) on addition of biotic interactions. This reduction in AUC may be influenced by the limited occurrence data for many of the species, especially given the large geographical area (Supplementary Table SI). However, given the theoretical benefits of including biotic interactions, we expect that models taking into about networks of interactions will produce more reliable predictions of future impacts. This will be an important area for future research - the inclusion of biotic interactions in predictions of species’ distributions shows promise but more research is needed to understand how best to incorporate network analyses and to evaluate the benefits (or not) of doing so.

An alternative method for resolving the Bayesian network could be used to better represent biotic interaction strengths. We selected the OR method to resolve the Bayesian network, but the more complex SUM approach may prove to be better (Staniczenko et al. 2017). The posterior of the OR model has only three states: increased, decreased and no change; by contrast, the SUM model improves on this such that the presence of more dependencies results in a stronger interaction. This leads to multiple posterior states (small and large increases, small and large decreases, and no change) that would allow for more accurate representation of the impact of interacting species.

Finally, we note that SDM approaches are correlative and subject to several assumptions and uncertainties (Pearson and Dawson, 2003; Guisan and Thuiller 2005; Dormann 2007; Peterson et al. 2011), so future predictions should be viewed with caution. Still, the indicative trends in potential future distribution of *G. belina* are noteworthy and raise a flag for future conservation efforts to monitor and anticipate changes that could have important implications for human wellbeing (Ndlovu et al. 2019). We hope that by exploring how to improve these methods by incorporating biotic interactions, it will be possible in the future to anticipate biogeographic trends more reliably

## Supplementary Material

Table S1

## Figures and Tables

**Table 1:** Predicted suitable habitat area predicted under the non-network and network models under different climate scenarios in ‘000km^2^. Percentage change represents proportion of suitable habitat change compared to predicted present distribution area.

	Present	2041-2060 (% change)		2061-2080 (% change)	
		RCP 4.5	RCP 8.5	RCP 4.5	RCP 8.5
No network	1480	905 (-39)	1110 (-25)	1010 (-32)	1150 (-23)
Network	649	85.6 (-87)	95.3 (-85)	91.1 (-86)	86.3 (-87)

**Table 2:** Habitat fragmentation calculated as the number of patches per 1000km^2^. Percentage change represents fragmentation change to predicted present habitat fragmentation.

	Present	2041-2060 (% change)		2061-2080 (% change)	
		RCP 4.5	RCP 8.5	RCP 4.5	RCP 8.5
No network	0.0537	0.106 (+97)	0.11 (+105)	0.127 (+136)	0.136(+153)
Network	0.0688	0.0294 (-58)	0.0396 (-42)	16.7 (-93)	0.0249 (-64)

**Table 3:** Suitable habitat distribution losses in ‘000 km^2^, under RCP 4.5 and 8.5 as predicted by Bayesian network species distribution models. Percentage losses represent the proportion of habitat lost compared to predicted present distribution area.

	Present	2041-2060 (% change)		2061-2080 (% change)	
		RCP 4.5	RCP 8.5	RCP 4.5	RCP 8.5
Total	649	239 (-63)	311 (-52)	285 (-56)	227 (-65)
Zimbabwe	237	10.4 (-99)	4.08 (-98)	16.7 (-93)	0 (-100)
Botswana	121	1.03 (-96)	3.54 (-97)	3.53 (-97)	0.012 (-100)
South Africa	62.6	21.8 (-52)	37.6 (-40)	37.1 (-41)	55.7 (-11)

## Data Availability

R code for resolving the Bayesian network and condensing distributions can be found at: https://git.io/JOKFx. Climate data are available from CHELSA (https://chelsa-climate.org/). GIBF data used in this study can be accessed as a derived dataset here: https://doi.org/10.15468/dd.z4qvef

## Highlights

- The mopane worm is an important source of food for people across southern Africa, yet insect declines driven by land use and climate change could threaten this important ecosystem service
- We modelled the potential future distribution of mopane worm based on abiotic drivers as well as biotic interactions with food and predator species
- The incorporation of biotic interactions using Bayesian networks represents an important step toward more realistic estimates of future species ranges
- Our models demonstrate the potential for substantial contraction of the range of mopane worm, which would likely have negative impacts on the health of people in rural communities
- Our study demonstrates a methodological advance in modelling species distributions and reveals how environmental change has potential to disrupt the benefits that nature provides for people
