# Supplementary material for: Potential decline in the distribution and food provisioning services of the mopane worm (Gonimbrasia belina) in southern Africa
Source: Front Biogeogr. Author manuscript; Available in PMC 2023 Sep 7. (PMC7615040; doi:10.21425/F5FBG59408)
Supplement: Table S1 [file EMS186861-supplement-Table_S1.pdf]

Frontiers of Biogeography Issue 15.2

Article: Potential decline in the distribution and food provisioning services of the mopane worm (Gonimbrasia belina) in southern Africa

David Yanliang Shen, Henry Ferguson-Gow, Vivienne Groner, Thinandavha Caswell Munyai, Rob Slotow, Richard Pearson

Supplementary Material

Supplementary Table S1: All species identified to have biotic interactions with *G. belina*.

Species	Interaction type	Included?	Source	GBIF records downloaded
		Birds		
Anthoscopus caroli	Predator	Yes	Styles (1995)	864
Bubalornis niger	Predator	Yes	Styles (1995)	2751
Bucorvus leadbeateri	Predator	Yes	Styles (1995)	2453
Burhinus capensis	Predator	Yes	Styles (1995)	6216
Campethera abingoni	Predator	Yes	Styles (1995)	4765
Chrysococcyx caprius	Predator	Yes	Styles (1995)	8415
Clamator levaillantii	Predator	Yes	Styles (1995)	1552
Coracias caudata	Predator	No	Styles (1995)	
Coracias naevia	Predator	No	Styles (1995)	
Corythaixoides concolor	Predator	Yes	Styles (1995)	8943
Creatophora cinerea	Predator	Yes	Styles (1995)	4672
Dendropicos namaquus	Predator	Yes	Styles (1995)	2656
Denropicos fuscescens	Predator	No	Styles (1995)	
Dircrurus adsimilis	Predator	No	Styles (1995)	
Eurocephalus anguitimens	Predator	Yes	Styles (1995)	2375
Gymnoris superciliaris	Predator	Yes	Styles (1995)	3290
Indicator indicator	Predator	Yes	Styles (1995)	3007
Lamprotornis nitens	Predator	Yes	Styles (1995)	11457
Lanius collurio	Predator	No	Styles (1995)	
Lybius torquatus	Predator	Yes	Styles (1995)	9021
Malaconotuc blanchoti	Predator	No	Styles (1995)	
Muscicapa striata	Predator	No	Styles (1995)	
Oriolus larvatus	Predator	Yes	Styles (1995)	7408
Passer griseus	Predator	Yes	Styles (1995)	359
Prinia subflava	Predator	Yes	Styles (1995)	8154
Pycnonotus barbatus	Predator	Yes	Styles (1995)	9432
Sagittarius serpentarius	Predator	Yes	Styles (1995)	4643
Sylvietta rufescens	Predator	Yes	Styles (1995)	8706
Telophorus sulfureopectus	Predator	Yes	Styles (1995)	1929
Tockus flavirostris	Predator	No	Styles (1995)	
Tockus nasutus	Predator	Yes	Styles (1995)	6144
Trachyphonus vaillantii	Predator	Yes	Styles (1995)	8875

Supplementary table 1 continued on next page

Continuation of supplementary table 1

Species	Interaction type	Included?	Source	GBIF records downloaded
Turdoides jardineii	Predator	Yes	Styles (1995)	5532
Turdus libonyana	Predator	Yes	Styles (1995)	4884
		Mammals		
Loxodonta africana	Competitive herbivore	Yes	De Nagy Koves Hrabar (2007)	2788
		Parasitoid wasps		
Mescocomys pulchriceps	Parasitoid	No	Van den Berg (1971)	
Eupelmus urozonus	Parasitoid	No	Van den Berg (1971)	
		Trees		
Brachystegia boehmii	Food tree	No	Mughogho and Munthali (1995)	
Brachystegia manga	Food tree	No	Mughogho and Munthali (1995)	
Brachystegia mapronfolia	Food tree	No	Mughogho and Munthali (1995)	
Brachystegia speciformis	Food tree	No	Mughogho and Munthali (1995)	
Brachystegia stipulata	Food tree	No	Mughogho and Munthali (1995)	
Colophospermum mopane	Food tree	Yes	Taylor and Moss (1982)	211
Diospyros mespiliformis	Food tree	Yes	Akinnifesi et al. (2008)	53
Diplorrhynchus condylocarpon	Food tree	No	Mughogho and Munthali (1995)	
Julbernardia globiflora	Food tree	Yes	Mughogho and Munthali (1995)	25
Julbernardia paniculata	Food tree	No	Mughogho and Munthali (1995)	
Pterocaprus angolensis	Food tree	No	Mughogho and Munthali (1995)	
Sclerocarya birrea	Food tree	Yes	Akinnifesi et al. (2008)	135
Terminalia sericea	Food tree	Yes	Mughogho and Munthali (1995)	98
Uapaca kirkiana	Food tree	Yes	Akinnifesi et al. (2008)	22
